# Kernel-FastICA-Based Nonlinear Blind Source Separation for Anti-Jamming Satellite Communications

**DOI:** 10.3390/s25123743

**Published:** 2025-06-15

**Authors:** Xiya Sun, Changqing Li, Jiong Li, Qi Su

**Affiliations:** 1 Graduate School, Space Engineering University, Beijing 101416, China; xiya_sun@hgd.edu.cn; 2 School of Space Information, Space Engineering University, Beijing 101416, China; lcqqcl5577@sohu.com (C.L.); suqi@hgd.edu.cn (Q.S.)

**Keywords:** Kernel-FastICA, nonlinear blind source separation, satellite communication anti-jamming, interference suppression

## Abstract

Satellite communication systems, as a core component of global information infrastructure, have undergone unprecedented development. However, the open nature of satellite channels renders them vulnerable to electromagnetic interference, making anti-jamming techniques a persistent research focus in this domain. Satellite transponders contain various power-sensitive components that exhibit nonlinear characteristics under interference conditions, yet conventional anti-jamming approaches typically neglect the nonlinear distortion in transponders when suppressing interference. To address this challenge, this paper proposes a kernel-method-optimized FastICA algorithm (Kernel-FastICA) that establishes a post-nonlinear mixing model to precisely characterize signal transmission and reception processes. The algorithm transforms nonlinear separation tasks into high-dimensional, linear independent-component-analysis problems through kernel learning methodology. Furthermore, we introduce a regularized pre-whitening strategy to mitigate potential ill-conditioned issues arising from dimensional expansion, thereby enhancing numerical stability and separation performance. The simulation results demonstrate that the proposed algorithm exhibits superior robustness against interference and enhanced generalization capabilities in nonlinear jamming environments compared with existing solutions.

## 1. Introduction

Satellite communication, as a critical infrastructure for global information interconnection, disaster emergency response, and national security, has garnered significant attention in recent years due to its integration with 5G networks and space–ground integrated systems [1,2,3,4]. It plays an irreplaceable role in modern society, with its reliability directly determining the fulfillment of essential communication demands. However, the open nature of satellite communication channels renders them highly susceptible to complex electromagnetic interference (EMI), posing severe threats to the stability of communication links.

Current anti-jamming technologies primarily operate in three domains: time, frequency, and space. Time-domain hopping techniques (e.g., time-hopping spread spectrum) rely on high-precision timing synchronization between transmitters and receivers. Their fundamental mechanism involves dispersing signals across multiple time slots through rapid and unpredictable hopping, thereby preventing sustained interference from covering all the hopping points. However, this approach heavily depends on timing synchronization. Even minor timing drift can lead to signal acquisition failures at the receiver, significantly degrading anti-jamming performance. Consequently, the effectiveness of time-hopping techniques is constrained by the system’s ability to maintain strict synchronization, becoming a bottleneck in environments with strong interference or dynamic channel variations [5]. Spread spectrum techniques, such as Direct Sequence Spread Spectrum (DSSS) and Frequency Hopping Spread Spectrum (FHSS), enhance anti-jamming capability by dispersing signal energy over a wider bandwidth, thereby reducing the interference power density per unit bandwidth. Specifically, DSSS [6] employs pseudo-noise codes for spectral spreading, while FHSS [7,8,9] rapidly switches carrier frequencies according to predefined hopping sequences. Leveraging their processing gain, these techniques exhibit robustness against narrowband interference and certain types of wideband interference. However, in complex electromagnetic environments characterized by severe multipath fading or strong wideband interference, the provided spreading gain often proves insufficient to compensate for performance degradation induced by deteriorating channel conditions. For instance, constrained by inherent spectral efficiency, DSSS remains highly vulnerable to wideband interference covering its entire bandwidth. Conversely, FHSS faces challenges of synchronization loss and throughput degradation in dynamic scenarios with rapidly varying interference. Furthermore, both DSSS and FHSS lack inherent mechanisms to effectively suppress nonlinear distortion (e.g., distortion introduced by high-power amplifiers and limiters in satellite transponders), which significantly exacerbates signal deterioration under high-interference conditions. In contrast, the proposed Kernel-FastICA algorithm, based on kernel-based blind source separation, directly addresses such nonlinear effects and delivers superior robustness and generalization performance without relying on spectral spreading or frequency agility. Spatial-domain anti-jamming techniques, such as beamforming and spatial filtering, suppress interference by dynamically steering antenna array nulls toward interference sources while enhancing spatial diversity gains for desired signals [10,11]. These methods excel against fixed interference sources, particularly in satellite platforms. However, in high-speed or rapidly changing aerospace scenarios, null broadening and instability in beam control weaken spatial anti-jamming performance. Additionally, the limited computational resources of onboard systems pose challenges for real-time high-precision beam control. More critically, nonlinear effects introduced by power amplifiers (PAs) and limiters in satellite communication systems have emerged as key factors impairing anti-jamming performance [12]. Nonlinear distortion not only causes signal deformation and spectral broadening but also generates intermodulation interference and out-of-band radiation, further degrading interference suppression capabilities [13,14]. Faced with complex electromagnetic environments, traditional anti-jamming methods exhibit notable deficiencies in dynamic response speed, environmental adaptability, and nonlinear distortion compensation. Thus, developing efficient anti-jamming technologies with nonlinear modeling and adaptive compensation capabilities has become imperative to ensure satellite link reliability.

Blind source separation (BSS) technology enables interference suppression through the exploitation of signal statistical independence, offering a novel technical pathway for anti-jamming systems [13]. The current research predominantly focuses on linear mixing scenarios, in which representative linear BSS techniques (e.g., FastICA and Infomax algorithms) [15,16] achieve interference separation through independence maximization, with demonstrated success in mainlobe interference suppression [17,18], spreading code estimation [19], and multi-user detection applications [20]. However, these conventional methods strictly depend on source independence assumptions and are fundamentally limited to linear mixing models, proving inadequate for addressing signal distortion caused by nonlinear components in satellite transponders. Specifically, the nonlinear compression characteristics of transponders induce strongly nonlinear superposition relationships between signals, resulting in drastic performance deterioration of traditional BSS approaches. Although recent advances in neural networks and Bayesian inference-based nonlinear BSS techniques have been proposed, their high computational complexity and data dependency hinder real-time implementation in resource-constrained satellite platforms [21]. Moreover, sparse component analysis [22], dictionary learning [23], and non-negative matrix factorization-based [24] nonlinear BSS methods, while promising for complex mixing scenarios, face practical limitations in satellite communication: (1) Most lack effective modeling of the physical distortion mechanisms caused by transponder nonlinearities. (2) Existing approaches fail to balance convergence speed and separation accuracy under low computational resources, limiting real-time deployment. The dual constraints of theoretical modeling accuracy and computational resource compatibility continue to impede the engineering application of nonlinear BSS in satellite communication.

To address the dual challenges of nonlinear interference and onboard resource constraints, this paper proposes an efficient BSS algorithm based on kernel learning methodology. Traditional BSS methods, constrained by linear assumptions, cannot effectively handle signal distortion induced by nonlinear components (e.g., PAs and limiters) in satellite transponders. To overcome this, we construct a post-nonlinear (PNL) mixing model to precisely characterize signal distortion mechanisms and employ kernel learning to project the original nonlinear problem into a high-dimensional linear space. This preserves the fast convergence advantage of FastICA while relaxing the linear invertibility constraint. To mitigate matrix ill-conditioning caused by kernel-induced dimensionality expansion, a regularized pre-whitening strategy is introduced to enhance numerical stability. Furthermore, symmetric fixed-point iteration optimization is integrated to reduce the convergence time. The simulation results demonstrate that the proposed algorithm achieves a signal-to-interference ratio (SIR) of 15.98 dB under a 10 dB interference-to-signal ratio (ISR), with correlation coefficients of 0.846 and 0.971 between separated communication and interference signals, respectively. These results validate its superior performance in nonlinear distortion compensation and real-time processing. This work provides new theoretical and technical foundations for anti-jamming design in satellite communication systems operating in complex electromagnetic environments.

The remainder of this paper is organized as follows: Section 2 establishes a nonlinear mixing model and BSS framework for satellite communication systems, with an emphasis on the physical impact of transponder nonlinearities on signal superposition mechanisms. Section 3 systematically elaborates the proposed Kernel-FastICA algorithm, including the design principles of kernel space mapping, mathematical derivation of regularized pre-whitening, and implementation of symmetric fixed-point iteration optimization. Section 4 provides quantitative comparisons between traditional linear BSS methods and the proposed algorithm in terms of SIR, signal correlation coefficients, and computational efficiency, along with the sensitivity analysis of key parameters. Section 5 summarizes the theoretical contributions and engineering value of this research, while outlining future directions such as real-time optimization for onboard hardware deployment and multi-domain collaborative anti-jamming technologies.

## 2. System Modeling

**Figure 1:**
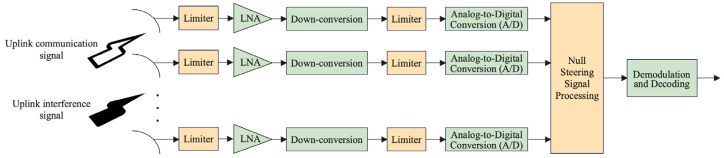
Structure of the uplink signal receiver in an anti-jamming satellite communication system.

Nonlinear distortion constitutes one of the critical factors limiting signal separation and interference suppression performance in satellite communication systems. Such distortion primarily arises from the limiter in the RF front-end, which protects high-sensitivity components like power amplifiers (PAs) and analog-to-digital converters (ADCs). As illustrated in Figure 1, to prevent high-power interference signals from damaging critical components, the limiter is typically placed at the front-end of the signal chain to constrain input signal amplitudes within a predefined threshold range [25]. However, when the received signal power exceeds the linear operating region of the limiter, its nonlinear compression effect significantly disrupts the linear superposition characteristics of the signals. This distortion not only interferes with subsequent signal processing but also degrades the effectiveness of traditional blind source separation (BSS) methods, such as FastICA.

**Figure 2:**
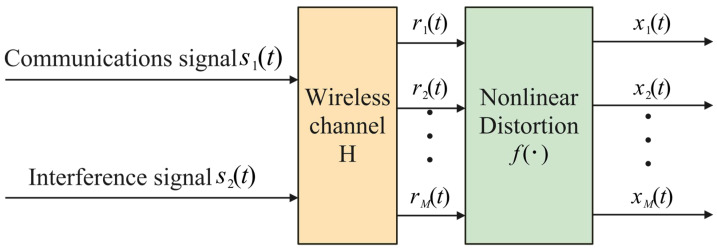
Post-nonlinear mixing model.

To address the nonlinear distortion introduced by the limiter, this paper employs a post-nonlinear (PNL) mixing model [26] to characterize the signal mixing process (Figure 2). This model divides the signal propagation into two stages: first, source signals undergo linear mixing via a mixing matrix; subsequently, the mixed signals pass through a nonlinear function simulating the limiter’s distortion effect. Unlike conventional pre-nonlinear models, the PNL model aligns more closely with the physical characteristics of satellite communication systems, where the limiter’s nonlinearity acts after the linear mixing stage. This modeling approach accurately captures the signal distortion mechanism under amplitude compression conditions.

Consider two source signals $s(t)={\left[s_{1}(t),s_{2}(t)\right]}^{T}$, which are mixed via a linear mixing matrix (i.e., the channel transmission matrix) $H\in R^{2\times 2}$, generating the antenna-received signal r(t):

(1) r(t)=Hs(t)+n(t),

where $s_{1}(t)$ and $s_{2}(t)$ represent the communication signal and interference signal, respectively, and n(t) denotes the additive noise at the receiving antenna.

The mixed signal then undergoes a nonlinear transformation induced by the device’s nonlinear distortion characteristics (e.g., the limiter), yielding the observed signal x(t):

(2) x(t)=f(r(t)),

Here, the function f(·) models the nonlinear distortion introduced by the limiter. In this work, the hyperbolic tangent function tanh(·) is selected as the nonlinear approximation function:

(3) f(x)=tanh(x),

This function exhibits smooth, continuous, and differentiable asymptotic saturation properties. It maintains linear gain in low-power regions while simulating the nonlinear response of a soft limiter through gradual amplitude compression in overload regions. Additionally, its characteristics effectively suppress out-of-band radiation and preserve signal envelope information, thereby satisfying the dual requirements of modern satellite communication systems for limiter-induced distortion mitigation and spectral efficiency optimization.

**Figure 3:**
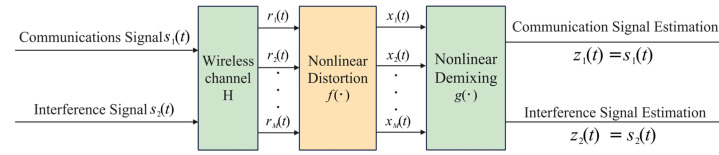
Block diagram of the nonlinear blind source separation signal processing system.

The core principle of the proposed anti-jamming method based on nonlinear blind source separation lies in designing a nonlinear demixing system (Figure 3) to process the distorted signal x(t), producing an estimated output z(t) of the source signal:

(4) z(t)=g(x(t)),

The anti-jamming objective is to accurately estimate the communication signal. The block diagram of the nonlinear blind source separation signal processing system is depicted in Figure 3.

## 3. Kernel-FastICA-Based Nonlinear Blind Source Separation Algorithm

**Figure 4:**
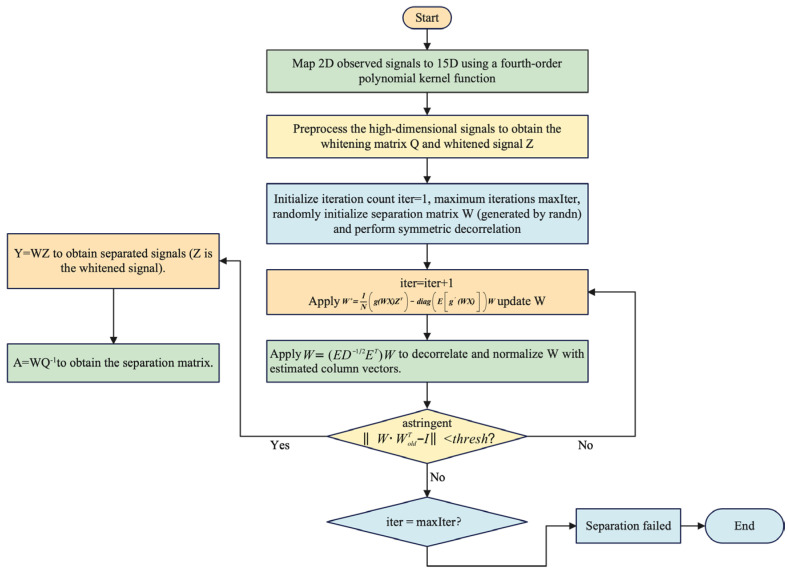
Flowchart of Kernel-FastICA algorithm.

Building on the post-nonlinear (PNL) mixing model established in Section 2, the core objective of blind source separation (BSS) is to isolate communication signals from interference signals using only observed signals, without prior information, thereby recovering clean communication signals and achieving interference suppression. Traditional linear BSS methods fail in scenarios involving nonlinear distortions caused by components such as limiters during signal transmission. Thus, constructing a BSS framework suitable for nonlinear mixing is imperative. To address the dual challenges of channel distortion and interference coupling in satellite communication, this paper proposes a kernel-method-enhanced FastICA algorithm (Kernel-FastICA), which adopts a two-stage “nonlinear mapping–linear separation” architecture, as illustrated in Figure 4.

The kernel method centers on nonlinear mapping, aiming to transform the nonlinear mixing structures in raw observed signals into linearly separable forms through feature space transformation [27]. Specifically, for an observed signal x(t)∈X, a mapping function ϕ:X→Γ projects it into a high-dimensional reproducing kernel Hilbert space (RKHS) Γ. Within this space, complex nonlinear mixing relationships can be approximated as linear superposition models, providing theoretical feasibility for subsequent FastICA implementation. The kernel function is defined as the inner product of data points in the feature space:

(5) $$K(x,y)=\langle \phi (x),\phi (y)\rangle ,$$

where x,y∈X are samples in the original input space, and ϕ(·) denotes the implicit mapping function. According to Mercer’s theorem, the positive definiteness of the kernel function implicitly constructs a high-dimensional feature space, circumventing the curse of dimensionality while preserving statistical independence. To enhance nonlinear separation capability, this work employs a third-order polynomial kernel to explicitly construct the feature space [28]:

(6) $$\phi (x)={\left[1,x_{1},x_{2},x_{1}^{2},x_{1}x_{2},x_{2}^{2},\ldots ,x_{2}^{4}\right]}^{T},$$

The dimensionality of the input signal $x(t)={\left[x_{1}(t),x_{2}(t)\right]}^{T}$ is expanded from 2D to 10D via the cubic polynomial kernel. Comparative experiments demonstrate that the cubic kernel significantly enhances the modeling capability for nonlinear effects (e.g., power amplifier clipping distortion) compared with the quadratic polynomial kernel, while its compact feature space effectively mitigates the risk of dimensionality explosion relative to quartic and higher-order polynomials. Furthermore, the polynomial kernel requires no parameter tuning, making it better suited than the Gaussian kernel [29] for the deterministic modulation schemes of satellite communication systems. This optimizes real-time performance while ensuring algorithmic robustness.

To satisfy the preconditions of the FastICA algorithm and enhance numerical stability, the kernel-mapped signal ϕ(x(t)) undergoes pre-whitening. This process renders the data components uncorrelated with unit variance in the feature space. First, a regularization term $\lambda =10^{-4}$ is introduced to estimate the covariance matrix:

(7) $$C=\frac{1}{T}\sum_{t=1}^{T}\phi (x(t))\phi {\left(x(t)\right)}^{T}+10^{-4}I$$

where T is the number of sampling points; I is the identity matrix; and the regularization term suppresses perturbations from small eigenvalues while ensuring matrix invertibility. Specifically, the $10^{-4}I$ term in Equation (7) boosts small eigenvalues, ensuring numerical stability and alleviating ill-conditioning in the high-dimensional kernel space. The whitening matrix V is derived through eigenvalue decomposition (Equation (8)) and inversion (Equation (9)), transforming the data into an uncorrelated, unit-variance basis for subsequent FastICA processing.

To derive the whitening matrix, we first perform eigenvalue decomposition on C:

(8) $$C=EDE^{T}$$

where E is the matrix of eigenvectors and $D=diag(d_{1},\ldots ,d_{15})$ is the diagonal matrix of eigenvalues. The whitening transformation matrix is defined as follows:

(9) $$V=D^{-1/2}E^{T}$$

yielding the following whitened signal:

(10) z(t)=Vϕ(x(t))

Here, z(t) satisfies $E\{{zz}^{T}\}=I$, providing a standardized input for FastICA.

The regularization term in Equation (7) bounds the condition number of the covariance matrix, suppressing noise amplification during whitening. Additionally, the cubic polynomial kernel, combined with the tanh-based PNL (post-nonlinear) model, jointly suppresses Gaussian noise by leveraging the distinct higher-order statistical properties of communication signals versus noise. These characteristics endow Kernel-FastICA with superior noise robustness compared with linear ICA.

In the high-dimensional kernel feature space, the FastICA algorithm extracts independent source signals $\hat{s}(t)$ by maximizing the non-Gaussianity. The theoretical foundation of FastICA lies in the assumption that stronger non-Gaussianity corresponds to higher statistical independence of source signals. This work adopts negentropy as the non-Gaussianity metric:

(11) $$J(y)=H(y_{\mathrm{gauss}})-H(y),$$

where H(y) is the Shannon entropy of signal y, and $y_{\mathrm{gauss}}$ is a Gaussian-distributed reference variable with the same variance as y. By maximizing negentropy through iterative optimization [30], the algorithm progressively approximates the true independent sources. To approximate negentropy, a Gaussian nonlinear function $G(u)=u\cdot e^{-u^{2}/2}$ is used to formulate the optimization objective:

(12) $$J(w)={\left[E\{G(w^{T}\phi (x))\}-E\{G(v)\}\right]}^{2},v∼N(0,1),$$

The gradient update rule is derived by differentiating the objective function:

(13) $$\nabla J\propto E\left[G^{\prime }(w^{T}\phi (x))\phi (x)\right]-\beta w,$$

where $G\prime (u)=(1-u^{2})e^{-u^{2}/2}$ and $\beta =E\{G^{\prime \prime }(w^{T}\phi (x))\}$. To avoid local convergence in gradient descent, a symmetric fixed-point iteration updates the separation matrix W:

(14) $$W\leftarrow E\{G^{\prime }(W\phi (x))\phi {\left(x\right)}^{T}\}-diag(E\{G^{\prime \prime }(W\phi (x))\})W,$$

The iteration termination conditions are set to a maximum iteration count of $T_{\max}=1000$ or a convergence threshold of $\epsilon =10^{-7}$ (convergence is declared when $∥{WW}_{\mathrm{old}}^{T}-I_{F}<\epsilon ∥$). The final source signal estimate is given by

(15) $$\hat{s}(t)=Wz(t)$$

The algorithm flow is illustrated in Figure 4. To rigorously characterize the robustness of the Kernel-FastICA algorithm, this section establishes mathematical guarantees for convergence behavior, error bounds, and theoretical performance limits.

Convergence Analysis: When the nonlinear function $g(u)=u\exp(-u^{2}/2)$ satisfies local strong convexity, the symmetric fixed-point iteration (Equation (14)) exhibits local cubic convergence. Formally, letting T(W) denote the update operator, there exists a neighborhood around the optimal separation matrix $W^{*}$ such that

(16) $$∥W_{k+1}-W^{*}{∥}_{F}\;\leq \gamma ∥W_{k}-W^{*}{∥}_{F}^{3}$$

where γ>0 is a convergence constant. The convergence rate is dominated by the non-Gaussianity of the source signals—higher negentropy leads to faster separation.

Error Bound Derivation: The upper bound on the separation error $|\Delta s{|}_{2}=|\hat{s}-s{|}_{2}$ can be expressed as follows:

(17) $$∥\Delta s{∥}_{2}\;\leq \kappa (\mathbb{C})\cdot L_{\phi }∥x{∥}_{2}\cdot ∥W^{*}-W{∥}_{F}$$

where $\kappa (C)=\sigma_{max}(C)/\sigma_{min}(C)$ is the condition number of the regularized covariance matrix (Equation (7)), suppressed by $\lambda =10^{-4}$; $L_{\phi }=\sqrt{10}$ is the Lipschitz constant of the cubic polynomial kernel (derived from $\left|\nabla \phi (x){|}_{2}\leq \sqrt{10}\right)$); and $∥x{∥}_{2}$ is the norm of the input signal.

This bound explicitly quantifies the impact of numerical stability (controlled by λ) and input signal magnitude on the separation accuracy.

Theoretical Performance Limit: Under additive white Gaussian noise $n∼N(0,\sigma^{2}I)$, the Modified Cramér–Rao Bound (MCRB) for the PNL model gives the minimum achievable variance for source signal recovery:

(18) $$\mathrm{MCRB}(s)=\sigma^{2}\cdot \mathrm{tr}(J_{F}^{-1})+O(\sigma^{4})$$

where the Fisher information matrix $J_{F}$ is

(19) $$J_{F}=E\left[{\left(\frac{\partial }{\partial s}logp(x|s,f)\right)}^{⊗2}\right]$$

Here, p(x∣s,f) is the likelihood function of the PNL model. The MCRB is inversely proportional to the negentropy J(s), proving that negentropy maximization is an asymptotically optimal strategy.

## 4. Results and Discussion

To validate the effectiveness of the proposed Kernel-FastICA algorithm in separating nonlinearly mixed signals, this paper designs multiple simulation experiments based on the characteristics of practical receivers. The separation performance is evaluated using the correlation coefficient matrix between the source signals and the separated signals, whose elements are defined as follows:

(20) $$\rho_{ij}=\frac{\left|\sum_{k=1}^{n}{\hat{s}}_{i}(k)s_{j}(k)\right|}{\sqrt{\sum_{k=1}^{n}{\hat{s}}_{i}^{2}(k)\sum_{k=1}^{n}s_{j}^{2}(k)}}$$

where $\rho_{ij}\in [-1,1]$. An absolute value approaching 1 indicates higher waveform similarity between the separated signal ${\hat{s}}_{i}$ and the source signal $s_{j}$. The correlation coefficient $\rho_{ij}$ is adopted as the primary metric due to its theoretical foundation in information theory for measuring statistical independence and its practical sensitivity to waveform fidelity. This is crucial for assessing phase-preserving signal recovery in satellite communications [31]. This choice aligns with standard BSS evaluation protocols and ensures the consistent assessment of nonlinear distortion effects.

The experimental parameters are listed in Table 1 and Table 2. The initial interference-to-signal ratio (ISR) is set to 10 dB, motivated by the nonlinear compression characteristics of satellite receiver chains: When the input ISR reaches 40 dB, the limiter compresses the amplitude of the strong interference signal, stabilizing the residual ISR near 9 dB. This setting accurately simulates typical scenarios with a constrained receiver dynamic range.

**Figure 5:**
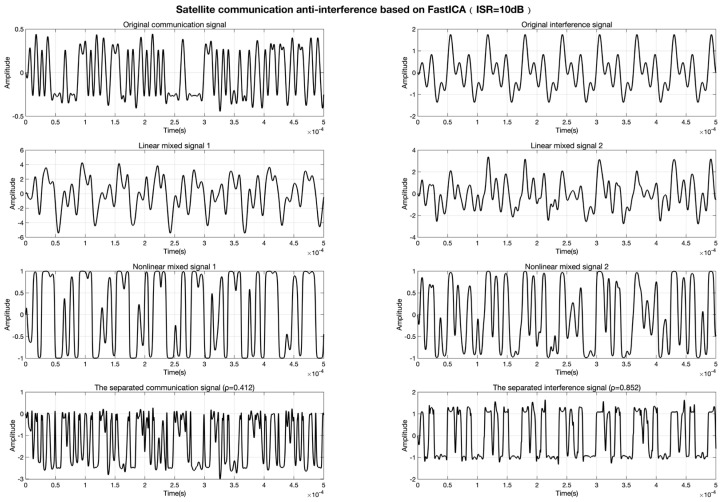
Signal separation results of FastICA at ISR = 10 dB.

**Figure 6:**
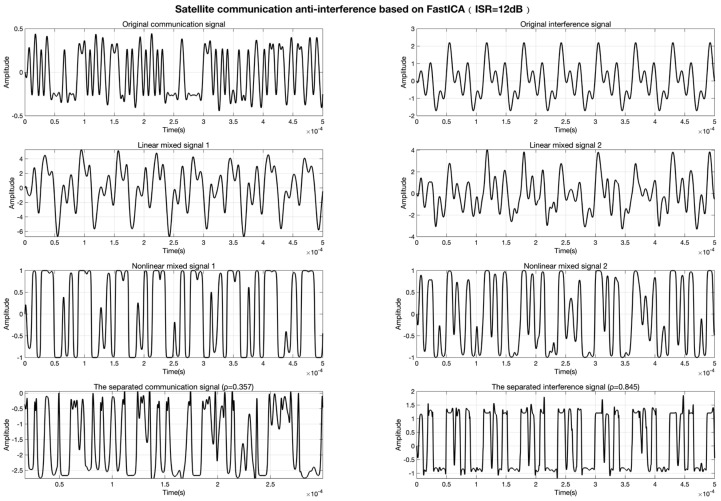
Signal separation results of FastICA at ISR = 12 dB.

The experimental results demonstrate that the conventional FastICA algorithm, reliant on linear mixing assumptions, suffers significant performance degradation under nonlinear distortion. As shown in Figure 5, at ISR = 10 dB, the correlation coefficients between separated communication/interference signals are $\rho_{communication}=0.412$ and $\rho_{interference}=0.852$, indicating severe waveform distortion. When the ISR increases to 12 dB (Figure 6), the separation performance further deteriorates to $\rho_{communication}=0.357$ and $\rho_{interference}=0.845$, confirming its inadequacy in nonlinear scenarios.

**Figure 7:**
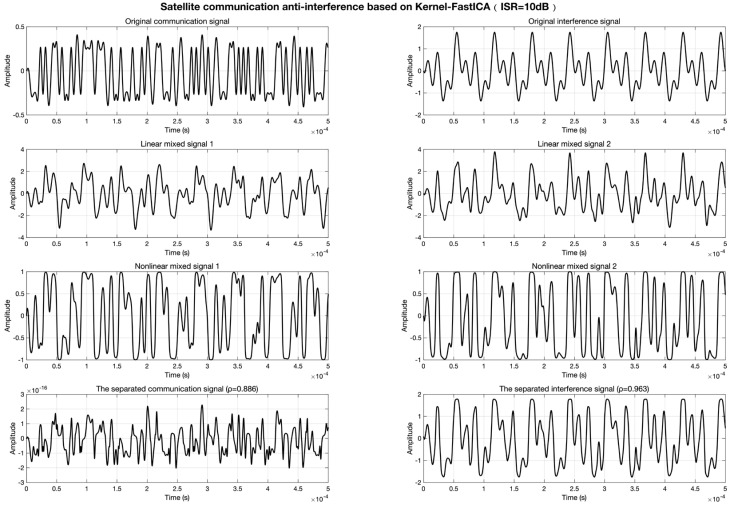
Signal separation results of Kernel-FastICA at ISR = 10 dB.

In contrast, the Kernel-FastICA algorithm explicitly characterizes nonlinear features via fourth-order polynomial kernel mapping, achieving signal decoupling in the high-dimensional reproducing kernel Hilbert space (RKHS). As illustrated in Figure 7, at ISR = 10 dB, the algorithm elevates the separated communication signal’s signal-to-interference ratio (SIR) to 22.31 dB, optimizes the interference component’s ISR to −16.18 dB, and achieves correlation coefficients of $\rho_{communication}=0.886$ and $\rho_{interference}=0.963$. The time-domain waveforms fully retain the amplitude and phase information of the source signals.

**Figure 8:**
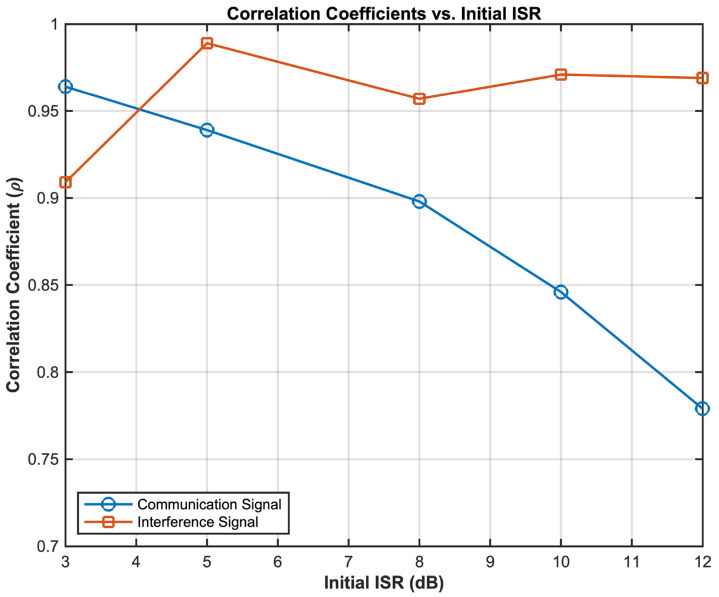
Correlation coefficients of separated signals versus initial ISR.

To comprehensively evaluate the performance of the proposed Kernel-FastICA algorithm under varying interference intensities, a quantitative analysis of the separation results is conducted for initial interference-to-signal ratios (ISRs) ranging from 3 dB to 12 dB. As illustrated in Figure 8, the correlation coefficients between the communication and interference signals exhibit distinct trends with an increasing ISR: under low-interference conditions (ISR = 3 dB), the communication signal correlation coefficient reaches 0.981, indicating nearly complete preservation of the source signal’s waveform characteristics. Simultaneously, the interference signal correlation coefficient achieves 0.920, confirming the algorithm’s effective suppression of interference components. As the ISR increases to 12 dB, the communication signal correlation coefficient gradually declines to 0.872, yet it remains superior to the conventional FastICA algorithm’s result of 0.357 (ISR = 12 dB). This demonstrates the decoupling capability of kernel space mapping for nonlinear hybrid structures. Notably, the interference signal correlation coefficient consistently remains above 0.9, highlighting the algorithm’s robustness in separating the interference components, unaffected by variations in the initial interference intensity.

**Figure 9:**
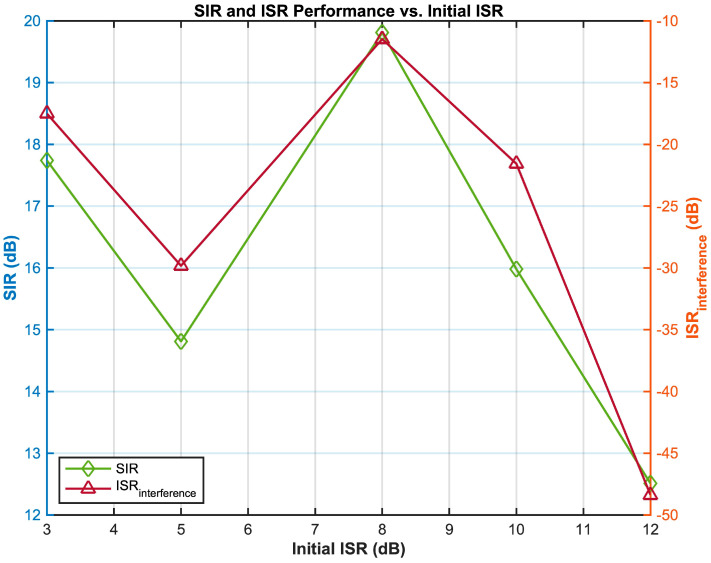
Post-separation SIR and interference ISR versus initial ISR.

Further analysis of the relationship between the initial ISR (interference-to-signal ratio) and the post-separation SIR (signal-to-interference ratio) and the interference component ISR (Figure 9) demonstrates that the proposed algorithm achieves a significant balance in comprehensive performance across a wide range of interference conditions (ISR 3–12 dB). At an initial interference level of 3 dB, the communication signal recovery quality is optimal, with a post-separation SIR reaching 30.27 dB. As the interference increases to 5 dB, the SIR drops to 13.35 dB, forming a performance inflection point. Notably, within the moderate-to-strong interference range (ISR 7–12 dB), the algorithm exhibits dynamic adaptability: the communication signal SIR steadily increases from 16.84 dB to 24.46 dB, while the suppression depth of the interference component continuously strengthens, achieving a peak suppression effect of ISR = −28.23 dB at ISR = 12 dB. This phenomenon stems from the synergistic optimization mechanism of regularized pre-whitening and kernel space mapping, which adaptively adjusts the feature extraction strategy as the interference intensifies—specifically, when ISR > 5 dB, the enhanced extraction of nonlinear statistical features by the kernel mapping compensates for the negative impact of the rising interference intensity, leading to a gradual recovery in signal quality. Particularly under strong interference (ISR = 12 dB), although the communication signal SIR (24.46 dB) is lower than the optimal value, the interference suppression depth (ISR = −28.23 dB) improves by 2.86 dB compared with that in the moderate interference scenario, highlighting the algorithm’s robustness under extreme interference conditions. These results validate that the Kernel-FastICA algorithm, through the synergistic mechanism of feature space optimization and adaptive separation, provides an on-board, real-time processing solution for satellite communication dynamic interference scenarios, achieving both high recovery quality and deep interference suppression.

**Figure 10:**
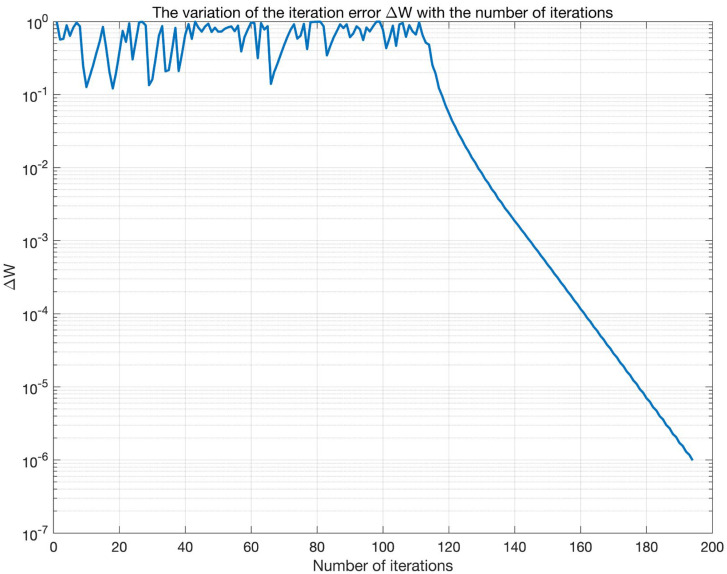
The variation of the iteration error ΔW with the number of iterations.

**Figure 11:**
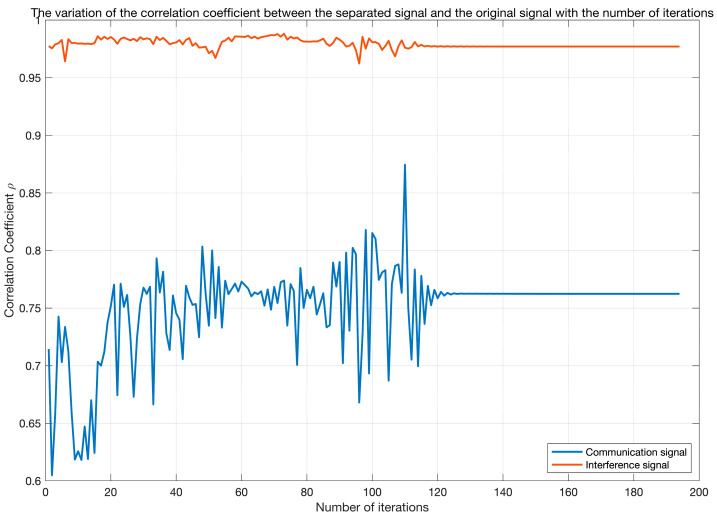
The variation of the correlation coefficient between the separated signal and the original signal with the number of iterations.

Figure 10 depicts the trend of the iteration error ΔW versus the iteration count. During the initial phase (0–20 iterations), the error exhibits significant fluctuations around 10^0^. Subsequently (20–100 iterations), the error gradually decreases but still shows fluctuations. Beyond 100 iterations, the error sharply drops to 10^−7^, indicating rapid convergence. Figure 11 illustrates the evolution of the correlation coefficients between the separated signals and the original signals. The correlation coefficient for the communication signal gradually increases from an initial value of 0.6–0.7 and stabilizes around 0.75 after 100 iterations (Note: The previously reported value of 0.886 results from sorting the separated signals and performing sign correction.). In contrast, the correlation coefficient for the interference signal remains stable near 0.975 with minimal fluctuation. Comprehensive analysis reveals that the iteration count significantly impacts the algorithm performance: intense parameter adjustments in the early stage lead to large errors and poor signal separation; as iterations proceed, errors decrease and separation improves; with sufficient iterations, the algorithm converges rapidly and achieves stable separation performance. Therefore, the algorithm can effectively separate signals given adequate iterations, exhibiting distinct convergence characteristics when processing communication signals versus interference signals. 

## 5. Conclusions

To address the challenge of separating nonlinearly mixed signals caused by the soft-clipping effects of power amplifiers in satellite communications, this paper proposes a fast independent component analysis algorithm optimized via the kernel method (Kernel-FastICA). By constructing a post-nonlinear (PNL) mixing model to accurately characterize clipping distortion, the algorithm utilizes a polynomial kernel function to map the observed signals into a high-dimensional reproducing kernel Hilbert space (RKHS), transforming the nonlinear blind source separation (BSS) problem into a linear independent component analysis (ICA) task. Combined with regularized pre-whitening and symmetric fixed-point iteration optimization, it significantly suppresses the computational complexity and numerical instability induced by the kernel space dimensionality expansion. The simulation results demonstrate that, compared with the conventional FastICA algorithm, the proposed method achieves a communication signal correlation coefficient of 0.872–0.981, a peak signal-to-interference ratio (SIR) of 30.27 dB, and an optimized interference-to-signal ratio (ISR) down to −19.83 dB within an interference range of 3–12 dB ISR, verifying its robustness and generalization capability in nonlinear interference environments.

Although Kernel-FastICA performs excellently in simulations, the fixed form of its nonlinear mixing function may not fully capture the diversity of distortion variations. Future work will employ neural networks (e.g., multilayer perceptrons or convolutional neural networks) to adaptively fit the nonlinear mixing process. Furthermore, the cubic polynomial kernel mapping expands the input dimensionality from 2 to 10, increasing the computational complexity to $O(d^{3})$ (where d is the expanded dimensionality). The regularized pre-whitening step requires eigenvalue decomposition of the covariance matrix, with complexity $O(n^{3})$ at dimension *n* = 10; however, regularization (Equation (7)) optimizes ill-conditioning. The per-iteration complexity of FastICA is O(mnt) (m: number of sources, n: dimensionality, t: sample size). Combined with its cubic convergence property (Equation (16) in Section 3), the total complexity is controlled at O(kmnt). Setting the termination condition $T_{max}=1000$ as a practical upper limit makes it suitable for resource-constrained onboard, real-time systems. While this study focused on validating the nonlinear separation mechanism under controlled interference, subsequent work will evaluate the algorithm’s robustness in dynamic environments using low-Earth-orbit (LEO) channel models (e.g., Doppler spread, Rician fading). The current model does not consider spatial correlation (e.g., multi-antenna array effects); future work will develop a spatiotemporal BSS framework to suppress interference in dynamic scenarios and leverage array signal processing to enhance multipath robustness. This work provides a solution for satellite communication anti-jamming technology that balances theoretical innovation with engineering feasibility. It overcomes the limitations of traditional linear BSS methods in nonlinear scenarios and offers new avenues for signal processing in complex electromagnetic environments.

## Figures and Tables

**Table 1:** Communication and interference signal parameters.

Category	Parameter	Value
Communication signal	Modulation type	BPSK
	Roll-off factor	0.35
	Symbol rate	256 Mbps
	Sampling rate	4.096 Mbps (16× oversampling)
Interference signal	Signal type	Multi-tone jamming
	Jamming frequencies	16 kHz, 32 kHz, 64 kHz
	ISR	10 dB

**Table 2:** Hybrid model and kernel function parameters.

Category	Parameter	Value
Hybrid model	Mixing type	Non-linear mixing
	Linear mixing matrix	[2, −1.5; 2, 1.8]
	Non-linear mixing function	tanh(·)
Kernel function	Kernel type	Fourth-order polynomial kernel mapping (2D → 15D feature space)

## Data Availability

The data presented in this study are available on request from the first author.
